# Socioeconomic differences in prevalence of biochemical, physiological, and metabolic risk factors for non-communicable diseases among urban youth in Delhi, India

**DOI:** 10.1016/j.pmedr.2018.08.006

**Published:** 2018-08-09

**Authors:** M. Arora, C. Mathur, T. Rawal, S. Bassi, R. Lakshmy, G.P. Nazar, V.K. Gupta, M.H. Park, S. Kinra

**Affiliations:** a Health Promotion Division, Public Health Foundation of India, Plot no. 47, Sector 44, Institutional Area Gurgaon, Haryana 122002, India; b Indian Institute of Health Management and Research University, Jaipur, Rajasthan 302029, India; c Department of Bio-chemistry, All India Institute of Medical Sciences, Ansari Nagar, New Delhi 110029, India; d Department of Health Services Research and Policy, Faculty of Public Health and Policy, London School of Hygiene and Tropical Medicine, UK; e Department of Non-communicable Disease Epidemiology, London School of Hygiene & Tropical Medicine, Keppel Street, London WC1E 7HT, UK

**Keywords:** Non-communicable disease, Risk factor, Socioeconomic status, Schools, India

## Abstract

This study examined whether the distribution of biochemical, physiological, and metabolic risk factors for non-communicable diseases (NCDs) among children and youth in urban India vary by socioeconomic status (SES). Data were derived from a cross-sectional survey of students enrolled in the 2nd and 11th grades in 19 randomly selected schools in Delhi (*N* = 1329) in 2014–15. Mixed-effect regression models were used to determine the prevalence of risk factors for NCDs among private (higher SES) and government (lower SES) school students. After adjusting for age, gender, and grade we found the percentage of overweight (13.16% vs. 3.1%, *p* value < 0.01) and obese (8.7% vs. 0.3%, *p* value < 0.01) students was significantly higher among private relative to government school students. Similarly, significantly higher percentage of private school students had higher waist circumference values (7.72% vs. 0.58%, *p* value < 0.01) than government school students. Furthermore, similar trend was observed across schools in the distribution of other NCD risk factors: raised blood pressure, raised total cholesterol, and low-density lipoprotein. Surprisingly, despite a higher prevalence of all risk factors, significantly higher percentage of private school students had adequate/ideal levels of high-density lipoprotein. Overall, the risk profile of private school students suggests they are more vulnerable to future NCDs.

## Highlights

• The prevalence of NCD risk factors is on the rise among children in India.
• SES differences in NCD risk factor distribution were studied in Delhi children.
• Private school students (high SES) experienced higher NCD risk vs. Govt. schools.
• Overweight/obesity, hypertension, dyslipidemia were higher in private schoolers.
• Tailored, school-based health promotion interventions are needed in India.

## 1 Introduction

With over 39 million deaths annually, non-communicable diseases (NCDs) account for 72% of global deaths (IHME, 2018). Moreover, the majority of NCD deaths occur in low-income and middle-income countries (WHO, 2018a; WHO, 2014) posing inequitable, health and economic burdens on individuals, societies, and health systems (WHO, 2011). NCDs that were more typically encountered in the West, appear to be rising in India, as it undergoes tremendous economic and epidemiologic transitions. NCDs, especially cardiovascular diseases, chronic obstructive pulmonary disease, cancers, diabetes mellitus and mental health disorders have emerged as major public health challenges for India (Mohan et al., 2011). Many of the NCD-attributable deaths occur in the most productive years of adult life as a consequence of risky behaviors acquired in youth.

In this era of globalization, India is experiencing an ever-increasing influence of Western culture, which is especially appealing to and accessible among populations belonging to higher socioeconomic status (SES) in this context (Singh, 1996). A lifestyle which promotes behaviors that escalate the risk for chronic diseases. Therefore, understanding the social class gradient in NCD risk factors is extremely important in halting the NCD epidemic in India. Pre-adolescence and adolescence represent a time for increased susceptibility to initiation and formation of long-term health behaviors (Perry, 2000). Behaviors such as unhealthy diet and physical inactivity could lead to raised blood pressure, blood glucose, blood cholesterol, and obesity, increasing their risk for NCDs. Most importantly, the multiplicity of the risk factors increases the risk of acquiring NCDs multifold (WHO, 2014).

Recent studies suggest that the prevalence of NCD risk factors is increasing rapidly among Indian youth. A study conducted with school children aged 10–16 years in Odisha (India) reported the prevalence of overweight and obesity to be 27.8% (Patnaik et al., 2015). Likewise, a second study conducted in Bangalore, India reported the prevalence of overweight and obesity among children aged 6–16 years to be 15.1% (Mishra et al., 2015). A study conducted in Kolkata, India with school children, aged 10–19 years found the prevalence of hypertension (systolic and/or diastolic blood pressure ≥ 95th percentile for gender, age and height) to be 10.1% (Maiti and Bandyopadhyay, 2016). Of note, in a study conducted with urban and rural school students at Karimnagar district, Telangana (India), 3.5% of students were reported to have diabetes mellitus (Kameswararao and Bachu, 2009). Additionally, in a study with type 2 diabetes patients in Chennai (India), 26% reported being diagnosed with diabetes mellitus before the age of 15 years (Amutha et al., 2012). However, to date, no published studies have explored the distribution of NCD risk factors among urban Indian youth, by SES.

As literature suggests that the prevalence of unhealthy behaviors among Indian youth is on the rise, it is important to gain a better understanding of the prevalence of risk behavior among this population. To address this gap in the literature, a school-based cross-sectional study was conducted to examine the distribution of NCD risk factors among youth in a large and socioeconomically diverse sample of private and government school students in Delhi. The study design includes two different types of schools- private and government which were used as a proxy for SES in this setting. Students from higher SES backgrounds generally study at the private schools, whereas those from lower SES backgrounds attend the government schools. Private schools generally cost several times more than the government schools, which either have a nominal fee or are offered free of cost (Sharma, 1999). Further, urban schools provide a sample of students of lower to higher SES and both sexes, whereas rural schools lack socioeconomic diversity.

## 2 Methods

### 2.1 Study design and setting

This school-based, cross-sectional study was conducted between 2013 and 2015, in 19 randomly selected (10 private and 9 government), co-educational senior secondary schools in New Delhi. These schools were randomly selected from the list of government and private schools governed by the Directorate of Education (DoE), Government of National Capital Territory (NCT) of Delhi (Directorate of Education, 2018)_._ The school type (private and government) was used as a proxy measure for SES in this study in accordance with earlier similar studies from India (Mathur et al., 2008)_._ Permissions for implementation of the study were obtained from the DoE and the School Health Scheme (SHS), Government of NCT of Delhi. Ethics approval for research involving human subjects for this study was obtained from the Public Health Foundation of India's (PHFI) Institutional Ethics Committee and Research Ethics Committee at London School of Hygiene and Tropical Medicine (LSHTM).

### 2.2 Study participants

A total of 1566 students (*n* = 729 from government and *n* = 837 from private schools) from the selected 19 schools were eligible to participate in the study. The sample size estimation was based on prevalence estimates of four major risk factors for NCDs (tobacco use, alcohol use, physical inactivity and overweight/obesity) in children from studies conducted in India (Tsering et al., 2010; Stigler et al., 2011; Mathur et al., 2008; Kameswararao and Bachu, 2009). An active informed consent procedure was followed in which informed consents from schools and parents along with students' assents were obtained after assuring confidentiality. The study sample was subdivided into two age groups: 6–7 years (*junior students from grade 2*) and 15–16 years (*senior students from grade 11*). Out of 1566 eligible students, 1329 (i.e., 85%) students who had complete data on all the anthropometric, biochemical, and physiologic measures, were included in our study.

### 2.3 Measures

The anthropometric, biochemical and physiological measurements of students were done by trained research staff, including a laboratory technician, using standardized protocols. Anthropometric measurements included height, weight and waist circumference (WC) measurement. Two readings for each student were obtained, using a protocol adapted to suit the Indian context (Lohman et al., 1988; Taylor et al., 2000). Omron Blood Pressure monitor (Model HEM-7120; with a pediatric cuff for junior students) was used to monitor the blood pressure of each student in sitting position (two readings per student, in resting condition and at an interval of 5 min). The biochemical analysis included lipid profile (total cholesterol (TC), high-density lipoprotein cholesterol (HDL-C) and low-density lipoprotein cholesterol (LDL-C) using Cholestech LDX (Model P/N: 412 00008) portable blood analyzer (Reis et al., 2006).

Students were grouped into four weight categories using the World Health Organization (WHO) age- and gender-specific Body Mass Index (BMI) growth references (WHO, 2018b, WHO, 2018c)_._These groups were underweight (BMI below −2 standard deviations [SD] in the WHO reference population), normal (between -2SD and 1SD), overweight (between 1SD and 2SD) and obese (more than 2SD).

Students whose WC values were more than age- and gender-specific 70th percentile cut-off were categorized as high WC and were considered to be at increased risk of NCDs (Khadilkar et al., 2014). The WC was measured using a non-stretchable measuring tape, at a level midway between the lower rib margin and iliac crest with participants in standing position; the measurements were done to the nearest 0.1 cm (Lohman et al., 1988).

Blood pressure categories were formed based on systolic blood pressure (SBP) and diastolic blood pressure (DBP) values by percentiles of height in boys and girls of age 3 to 18 years using cut-offs provided by Krishna et al. (2006). Normal, pre-hypertensive and hypertensive categories were defined for values of SBP and DBP both <90th percentile, between 90th to <95th percentile and ≥95th percentile, respectively.

Guidelines from the National Cholesterol Education Program for Children and Adolescents, USA were used to report TC, LDL-C, and HDL-C (NHLBI, 2012). For TC, the acceptable value was <170 mg/dl, the borderline value was between 170 and 199 mg/dl and the high value was ≥200 mg/dl. For LDL-C, the acceptable value was <110 mg/dl, the borderline value was between 110 and 129 mg/dl and the high value was ≥130 mg/dl. For HDL-C the acceptable value was >45 mg/dl, the borderline value was between 40 and 45 mg/dl and the low value was ≤40 mg/dl.

### 2.4 Statistical analysis

Mixed-effects regression models were used to investigate the prevalence of physiological, biochemical, and metabolic risk factors for NCDs among Indian youth. Mixed-effect models are used in cluster sample to minimize the Type I error rate and to ensure that the most conservative standard error is estimated (Raudenbush and Bryk, 2002). Models of the overall analysis were adjusted for age, gender and grade. Furthermore, the analyses were further stratified by gender and grade to better understand the distribution of these risk factors. The differences in the prevalence of NCD risk factors were considered to be statistically significant across government and private school students (and for the sub-group analyses) for a *p*-value < 0.05. All analyses were conducted using SAS for Windows (version 9.3; SAS Institute Inc.).

## 3 Results

The overall sample (*n* = 1329) consisted of 59% male students, 46.4% students from government schools and 43.2% students from grade two (Table 1). The prevalence estimates of physiological, biochemical, and metabolic risk factors for NCDs by school type are presented in Table 2. The percentage of overweight and obese students was significantly higher among private schools (13.16% overweight and 8.7% obese) compared with government schools (3.1% overweight, 0.3% obese), *p*-value < 0.01. Of note, the prevalence of underweight students in government schools was 3.2 times the prevalence of underweight in private schools (20.64% [95% CI 17.20–24.07] vs. 6.45% [95% CI 3.86–9.05], *p*-value < 0.01). The percentage of students within the normal weight category was almost equal across school type. Overall, these findings were mirrored across gender (Table 3) and grade (Table 4). Similarly, the percentage of students with high WC values was significantly higher among private school students compared with government school students (7.72% [95% CI 6.14–9.31] vs. 0.58% [95% CI 0.0–2.31], *p*-value < 0.01) (Table 2). Overall, a similar trend was seen across gender (Table 3) and grade (Table 4).

**Table 1:** Sample characteristics (Delhi: 2014–15).

Characteristic	Total students (*N* = 1329)	Government school students (*N* = 616)	Private school students (*N* = 713)
	N (%)	N (%)	N (%)
Gender			
Male	786 (59.14)	370 (60.06)	416 (58.35)
Grade			
2nd grade	574 (43.19)	232 (37.66)	342 (47.97)
Age (mean in years ± SD)		12.64 ± 4.77	12.25 ± 4.65

SD = standard deviation.

**Table 2:** Prevalence of biochemical, physiological and metabolic risk factors for NCDs in Delhi 2014–15, by school type (proxy for SES), adjusted.

	Government schools (*n* = 616)	Private schools (*n* = 713)	Ratio^b^	*p*-Value^a^
	Percentage (95% *CI*)	Percentage (95% *CI*)		
BMI				
Underweight	20.64 (17.20–24.07)	6.45 (3.86–9.05)	3.2	<0.01
Normal	75.66 (70.22–81.10)	71.56 (67.29–75.83)	1.06	0.21
Overweight	3.10 (0.00–7.14)	13.16 (9.87–16.44)	0.24	<0.01
Obese	0.30 (0.00–2.80)	8.7 (6.87–10.53)	0.03	<0.01

Waist circumference				
Normal	93.47 (97.69–100.0)	92.28 (90.69–93.86)	1.22	<0.01
High	0.58 (0.0–2.31)	7.72 (6.14–9.31)	0.08	<0.01

Blood pressure				
Normal	90.38 (85.62–95.14)	76.79 (72.62–80.65)	1.18	<0.01
Pre-hypertensive	5.26 (2.64–7.89)	10.42 (8.17–12.67)	0.5	<0.01
Hypertensive	4.48 (0.75–8.20)	12.74 (9.48–16.00)	0.35	<0.01

Total cholesterol				
Acceptable	97.66 (93.65–100.00)	84.74 (81.43–88.04)	1.15	<0.01
Borderline	2.50 (0.00–5.84)	12.60 (9.93–15.27)	0.2	<0.01
High	0.00 (0.00–0.00)	2.62 (1.42–3.82)	0	<0.01

High density lipoprotein (HDL)-cholesterol				
Acceptable	22.09 (16.83–27.36)	48.44 (44.29–52.00)	0.46	<0.01
Borderline	17.22 (12.99–21.45)	20.88 (17.64–24.12)	0.82	0.15
Low	60.75 (55.30–66.20)	30.67 (26.38–34.97)	1.98	<0.01

Low density lipoprotein (LDL)-cholesterol				
Acceptable	98.21 (95.27–100.00)	87.42 (85.15–89.70)	1.12	<0.01
Borderline	0.86 (0.00–3.24)	8.30 (6.47–10.13)	0.1	<0.01
High	0.99 (0.00–2.91)	4.29 (2.77–5.81)	0.23	0.01

a Estimates are generated from mixed-effects models adjusted for class/grade, gender, age.

b Ratio represents the prevalence ratio for the corresponding strata comparing students in government vs. private schools.

**Table 3:** Prevalence of biochemical, physiological and metabolic risk factors for NCDs in Delhi 2014–15, by school type (proxy for SES) and gender.

Girls (*n* = 543)				
Risk factors	Government schools (*n* = 246)	Private schools (*n* = 297)	Ratio^b^	*p*-Value^a^
	Percentage (95% *CI*)	Percentage (95% *CI*)		
BMI				
Underweight	14.97 (10.36–19.57)	5.83 (2.35–9.31)	2.57	<0.01
Normal	78.29 (71.30–85.28)	71.03 (65.75–76.31)	1.1	0.09
Overweight	6.14 (0.97–11.30)	14.40 (10.47–18.34)	0.43	0.01
Obese	0.71 (0.00–4.71)	8.85 (5.78–11.91)	0.08	<0.01

Waist circumference				
Normal	99.78 (97.26–100.0)	94.49 (92.37–96.62)	1.15	<0.01
High	0.22 (0.0–2.74)	5.51 (3.38–7.63)	0.04	<0.01

Blood pressure				
Normal	90.65 (84.23–97.07)	78.12 (73.36–82.87)	1.16	<0.01
Pre-hypertensive	7.76 (3.04–12.49)	11.16 (7.66–14.66)	0.69	<0.01
Hypertensive	1.67 (0.00–6.31)	10.75 (7.31–14.19)	0.15	<0.01

Total cholesterol				
Acceptable	95.56 (88.63–100.00)	83.41 (77.87–88.95)	1.14	<0.01
Borderline	4.55 (0.00–11.08)	14.70 (9.49–19.90)	0.31	0.02
High	0.00 (0.00–0.00)	1.86 (0.26–3.47)	0	0.12

High density lipoprotein (HDL)-cholesterol				
Acceptable	25.98 (18.30–33.66)	56.39 (50.59–62.20)	0.46	<0.01
Borderline	21.70 (14.88–28.51)	20.20 (15.05–25.35)	1.07	0.72
Low	52.10 (44.26–59.95)	23.41 (17.48–29.35)	2.22	<0.01

Low density lipoprotein (LDL)-cholesterol				
Acceptable	96.42 (91.52–100.00)	87.82 (84.11–91.53)	1.1	<0.01
Borderline	2.39 (0.00–6.51)	9.31 (6.19–12.42)	0.26	<0.01
High	0.89 (0.00–2.98)	2.74 (1.62–4.32)	0.32	<0.01


Boys (*n* = 786)				
	Government schools (*n* = 370)	Private schools (*n* = 416)		
BMI				
Underweight	24.94 (19.99–29.90)	7.75 (3.98–11.52)	3.22	<0.01
Normal	74.17 (65.91–76.71)	71.31 (67.21–81.13)	1.04	0.48
Overweight	0.47 (0.00–5.18)	12.07 (8.27–15.88)	0.04	<0.01
Obese	0.38 (0.00–3.71)	8.63 (6.13–11.14)	0.04	<0.01

Waist circumference				
Normal	99.22 (93.4–100.0)	90.65 (88.03–93.26)	1.32	<0.01
High	0.78 (0.0–9.6)	9.35 (6.74–11.97)	0.08	<0.01

Blood pressure				
Normal	90.05 (84.52–95.57)	74.09 (68.60–79.59)	1.21	<0.01
Pre-hypertensive	4.03 (0.79–7.28)	11.10 (7.79–14.42)	0.36	<0.01
Hypertensive	6.17 (0.67–11.67)	14.98 (9.63–20.32)	0.41	0.03

Total cholesterol				
Acceptable	99.58 (95.46–100.00)	85.46 (82.23–88.69)	1.16	<0.01
Borderline	0.59 (0.00–4.06)	11.19 (8.51–13.86)	0.05	<0.01
High	0.00 (0.00–0.00)	3.38 (1.96–4.80)	0	<0.01

High density lipoprotein (HDL)-cholesterol				
Acceptable	18.97 (12.50–25.44)	39.72 (12.50–25.44)	0.48	<0.01
Borderline	14.07 (8.74–19.40)	20.35 (16.24–24.45)	0.69	0.05
Low	66.72 (58.97–74.46)	39.83 (33.48–46.17)	1.67	<0.01

Low density lipoprotein (LDL)-cholesterol				
Acceptable	99.98 (96.40–100.00)	86.85 (84.08–89.63)	1.15	<0.01
Borderline	0.00 (0.00–0.00)	7.49 (5.31–9.66)	0	<0.01
High	0.96 (0.00–3.49)	5.64 (3.66–7.63)	0.17	<0.01

a Estimates are generated from mixed-effects models adjusted for class/grade, age.

b Ratio represents the prevalence ratio for the corresponding strata comparing students in government vs. private schools.

**Table 4:** Prevalence of biochemical, physiological and metabolic and metabolic risk factors for NCDs in Delhi 2014–15, by school type (proxy for SES) and grade.

2nd grade (*n* = 574)				
Risk factors	Government schools (*n* = 232)	Private schools (*n* = 342)	Ratio^b^	*p*-Value^a^
	Percentage (95% *CI*)	Percentage (95% *CI*)		
BMI				
Underweight	20.83 (16.31–25.35)	5.45 (1.76–9.13)	3.82	<0.01
Normal	76.90 (69.83–83.96)	75.01 (69.04–80.99)	1.02	0.7
Overweight	2.75 (0.00–6.23)	9.65 (6.81–12.48)	0.28	<0.01
Obese	0.00 (0.00–0.00)	9.98 (6.36–13.59)	0	<0.01

Waist circumference				
Normal	100.0 (92.88–100.0)	99.28 (91.49–100.0)	1.01	0.11
High	0.0 (0.0–7.12)	0.72 (0.00–8.51)	0	0.11

Blood pressure				
Normal	91.33 (84.59–98.06)	73.43 (67.25–79.62)	1.24	<0.01
Pre-hypertensive	3.28 (0.00–7.27)	13.22 (9.56–16.88)	0.25	<0.01
Hypertensive	5.58 (0.00–11.58)	13.41 (7.89–18.94)	0.42	0.07

Total cholesterol				
Acceptable	97.22 (92.24–100.00)	83.44 (79.21–87.67)	1.16	<0.01
Borderline	2.95 (0.00–7.53)	14.21 (10.33–18.09)	0.2	<0.01
High	0.00 (0.00–0.00)	2.43 (1.02–3.85)	0	0.03

High density lipoprotein (HDL)-cholesterol				
Acceptable	27.28 (19.71–34.87)	56.86 (50.45–63.26)	0.48	<0.01
Borderline	23.03 (17.05–29.01)	19.58 (14.62–24.55)	1.18	0.41
Low	49.63 (42.53–56.73)	23.44 (17.4–29.42)	2.12	<0.01

Low density lipoprotein (LDL)-cholesterol				
Acceptable	97.40 (93.71–100.00)	89.61 (86.53–92.68)	1.09	<0.01
Borderline	1.28 (0.00–4.36)	7.76 (5.19–10.32)	0.16	<0.01
High	1.32 (0.00–3.46)	2.64 (0.86–4.42)	0.5	0.38


11th grade (*n* = 755)				
	Government schools (*n* = 384)	Private schools (*n* = 371)		
BMI				
Underweight	18.13 (14.67–21.59)	4.69 (1.31–8.06)	3.86	<0.01
Normal	75.12 (69.45–80.79)	69.33 (63.94–74.71)	1.08	0.15
Overweight	4.65 (0.00–9.83)	16.74 (11.79–21.58)	0.28	<0.01
Obese	2.28 (0.00–5.49)	8.79 (5.77–11.81)	0.26	<0.01

Waist circumference				
Normal	98.91 (90.71–100.0)	88.66 (79.58–97.73)	1.54	<0.01
High	1.09 (0.0–9.29)	11.34 (2.27–20.42)	0.1	<0.01

Blood pressure				
Normal	90.44 (84.42–96.47)	78.68 (72.78–84.59)	1.15	0.01
Pre-hypertensive	5.51 (2.45–8.57)	7.32 (4.10–10.53)	0.75	0.44
Hypertensive	3.59 (0.42–7.13)	13.78 (10.10–17.46)	0.26	<0.01

Total cholesterol				
Acceptable	98.41 (94.51–100.00)	84.84 (81.15–88.53)	1.16	<0.01
Borderline	1.83 (0.00–4.66)	11.98 (9.21–14.75)	0.15	<0.01
High	0.00 (0.00–0.00)	3.09 (1.48–4.70)	0	0.01

High density lipoprotein (HDL)-cholesterol				
Acceptable	16.35 (11.82–20.89)	37.65 (33.21–42.10)	0.43	<0.01
Borderline	15.97 (11.59–20.36)	22.81 (18.51–27.11)	0.71	0.03
Low	67.38 (60.75–74.02)	39.95 (33.69–46.22)	1.69	<0.01

Low density lipoprotein (LDL)-cholesterol				
Acceptable	98.31 (95.19–100.00)	84.45 (81.38–87.51)	1.16	<0.01
Borderline	1.68 (0.00–4.33)	10.29 (7.68–12.90)	0.16	<0.01
High	0.74 (0.00–2.44)	5.22 (2.30–7.46)	0.14	<0.01

a Estimates are generated from mixed-effects models adjusted for gender, age.

b Ratio represents the prevalence ratio for the corresponding strata comparing students in government vs. private schools.

Our results for the prevalence of raised blood pressure reflect significantly higher percentage of students with pre-hypertension among private school students relative to government school students (10.42% [95% CI 8.17–12.67] vs. 5.26% [95% CI 2.64–7.89], *p*-value < 0.01) and with hypertension (12.74% [95% CI 9.48–16.00] vs. 4.48% [95% CI 0.75–8.20], *p*-value < 0.01) (Table 2). Following the trend of lower prevalence of risk factors among government school students, we found normal blood pressure to be 18% more prevalent in government school students compared with private school students. Likewise, across gender (Table 3) and grade (Table 4) higher prevalence of pre-hypertensive and hypertensive students among private schools than government schools was seen.

Similarly, the prevalence of high TC and high LDL-C was significantly higher among private school students relative to government school students (Table 2). None of the government school students had high TC, whereas 2.62% of private school students had high TC. The percentage of students with high levels of LDL-C was significantly higher among private schools than government schools (4.29% [95% CI 2.77–5.81] vs. 0.99% [95% CI 0.00–2.91], *p*-value < 0.01). Again, the findings were similar across gender and grade with private school students having a consistently higher prevalence of almost all the risk factors for NCDs measured in this study compared to government school students. The prevalence of low levels of HDL-C was higher among government school students than private school students (60.75% [95% CI 55.30–66.20] vs. 30.67% [95% CI 26.38–34.97], *p*-value < 0.01). These findings were similar across gender (Table 3) and grade (Table 4), albeit of varying magnitude.

## 4 Discussion

To the best of our knowledge, this is the first study to report on the distribution of multiple risk factors for NCDs in Indian school students, comparing low SES students (government schools) with those belonging to high SES (private schools). As indicated by the proportion of individuals in each risk factor category, an overall trend of clustering of risk factors in private school students was observed. As expected, these differences were mirrored in further sub-analyses by gender and grade.

Private schools had 4.24 times the prevalence of overweight students and 29 times the prevalence of obese students compared to government schools. Overall, 3.4% of students in government schools and 21.9% in private schools are either overweight or obese (Arora et al., 2012; Jagadesan et al., 2014). Our results corroborate and extend the findings from previous regional studies in India (Patnaik et al., 2015; Mishra et al., 2015; Khadilkar et al., 2014; Gupta et al., 2012). However, our results differ from findings from the developed world where overweight and obesity have been shown to be higher among low SES groups (Stamatakis et al., 2009). The sedentary lifestyle of private school students could have resulted in a high prevalence of overweight and obesity. Excess weight spells adverse metabolic consequences and is also an independent precursor for coronary heart disease, hypertension, breast cancer, and premature death (WHO, 2014).

In the present study, the prevalence of hypertensive students in private schools was 12.7% and in government schools it was 4.5%, which is in agreement with most studies in India (2.2% to 6.5%) (Maiti and Bandyopadhyay, 2016; Amritanshu et al., 2015; Charan et al., 2011). However, some studies have reported the prevalence of hypertension to be as high as 21.5% among adolescents (Sundar et al., 2013). These variations across school type could possibly be explained by differences in dietary habits specifically, excessive intake of ready-to-eat foods which are usually rich in sodium, an important contributing factor for raised blood pressure (Kumar et al., 2017).

With a higher prevalence of hypertension in higher SES group, the current study does not support the global trend, where hypertension is associated with lower SES group (Urrutia-Rojas et al., 2006; Kagura et al., 2016; Kulaga et al., 2010). The prevalence of students who had high WC values in our study was 0.6% and 7.7% for government and private school students, respectively. This was consistent with global estimates of 1.2% to 22.6% (Tailor et al., 2010). Policies and programs that encourage a healthy lifestyle (i.e., balanced diet and physical activity) should be enacted to combat NCD-related morbidity and mortality.

Interestingly, a higher prevalence of low HDL-C among students in government schools than in private schools was observed, suggesting that low SES youth are also vulnerable to possible CHD in the future. Among government school students, lower levels of HDL-C could be attributed to undernourishment and less than adequate intake of legumes, beans, whole grains, and high-fiber fruits. Additionally, refined carbohydrates and trans fats are contributory factors for low HDL-C levels (Gupta et al., 2012).

WHO in its sustainable development goal (SDG) 3 has set a target to reduce by one-third, premature mortality from NCDs by 2030 (WHO, 2018d). The Ministry of Health and Family Welfare (MoHFW), Government of India has also set a target to reduce premature mortality from NCDs by 25% by 2025 under its national action plan and monitoring framework for prevention and control for NCDs (MOHFW, 2013). To achieve these goals, it is important to focus on prevention of NCD risk factors through targeted health promotion interventions to specific vulnerable groups such as children and adolescents (Mohan et al., 2011). Various intervention programs in developed and developing countries like MARG in India (Shah et al., 2010), Trim and Fit in Singapore (Toh et al., 2002), and HealthKick (Draper et al., 2010) in South Africa have successfully demonstrated that children and adolescents can be educated about healthy lifestyle practices which can subsequently be helpful in reducing their risk of NCDs. The results of this study indicate that in the Indian context, NCD prevention interventions, and specifically healthy lifestyle interventions, need to be tailored for specific groups. The rising prevalence of some of the NCD risk factors among government school students as observed in our study suggests that they can benefit from overall healthy lifestyle interventions as well. Overall, these risk profiles should allow researchers to refine/modify their intervention strategies and target different subgroups, thus leveraging resources more effectively and developing more successful interventions that are sustained across time.

## 5 Strengths and limitations

The current study has some limitations that are worth noting. First, data on parent's occupation and family's caste/tribe was not collected in this study. This information, therefore, could not be used to determine a child's SES or to examine how these variables are related to the prevalence of NCD risk factors, independent of school type. However, school type is correlated with SES and can be used as a proxy for SES in this setting (Sharma, 1999). Future research should examine the effects of other measures of SES on risk factors for NCDs in the youth. Second, study findings are not necessarily representative of school children across India as a whole. As this study is cross-sectional by design, causality cannot be established and it is not possible to assess the trends and patterns of NCD risk factors over time. However, a large sample size ensures robust findings, and the study is representative of a large metropolitan Indian city. The findings should be replicated in other geographic populations. Most importantly, there has been no research on the distribution of NCD risk factors among Indian youth belonging to different SES groups, to date. Of note, the current study provides valuable surveillance data which could help guide policymakers develop and incorporate targeted strategies for NCD prevention in school health programs.

## 6 Conclusion

Students from affluent groups have a higher prevalence of NCD risk factors compared to the less privileged section of the society in the Indian context. There is a need for tailored, comprehensive school-based interventions to promote a healthy lifestyle to abate the increasing prevalence of NCD risk factors among children and adolescents. The most effective interventions would be those that transact across SES and address the social contexts of health behaviors. A healthier lifestyle may be an effective way to achieve robust health in individuals and even across generations.

## Conflict of interest

None.

## Funding

This work was supported by a Wellcome Trust Capacity Strengthening Strategic Award to the Public Health Foundation of India and a consortium of UK universities (Grant Number: WT084754/Z/08/Z). The funding agency was not involved in the study design; collection, analysis and interpretation of data; the writing of the manuscript; the decision to submit the manuscript for publication.
